# Parents are aware of the ototoxic effects of chemotherapy in paediatrics undergoing cancer treatment – Professional versus parental views: A pilot study

**DOI:** 10.4102/sajcd.v64i1.183

**Published:** 2017-02-27

**Authors:** Nomfundo F. Moroe, Kirstie Hughes

**Affiliations:** 1 Department of Speech Pathology and Audiology, School of Human and Community Development, University of the Witwatersrand, South Africa

## Abstract

**Background:**

The primary goal of chemotherapy is to cure cancer and its symptoms. Hence, in recent years, there has been an increase in cancer paediatric survival rate. However, there have also been adverse side effects such as ototoxic hearing loss because of chemotherapy. Therefore, this study aimed at exploring whether the parents of children undergoing chemotherapy are aware of ototoxic effects of chemotherapy.

**Methods:**

A non-experimental quantitative study was conducted to collect data through questionnaires, one for paediatric oncologists and the other for parents. A convenience sampling strategy was employed to recruit 11 paediatric oncologists and 7 parents from two public hospitals in Gauteng. The questionnaires were analysed quantitatively, using descriptive statistics.

**Results:**

About 55% of paediatric oncologists indicated informing parents about the ototoxic effects of chemotherapy. On the contrary, 71% of parents reported having been informed by paediatric oncologists about the possible hearing loss because of chemotherapy; however, 57% of the children are receiving a combination of cisplatin and cyclophosphamide despite being aware of their ototoxic nature.

**Conclusion:**

This study paves the way for qualitative studies to ascertain how parents are informed about the possible side effects such as hearing loss because of chemotherapy treatment. The mode in which parents are informed about the possible side effects related to chemotherapy is critical, considering that a high number of children are still receiving chemotherapeutic drugs that are directly linked to hearing loss.

## Introduction

Cancer is listed as one of the leading causes of mortality, not only internationally but also within the South African context (Mqoqi, Kellet, Sitas & Jula, 2004). In South Africa, cancer in the adult population is reportedly the highest in the continent (Mqoqi et al., 2004), while little to no data could be obtained for the paediatric population. It is a well-documented fact that chemotherapy, which is a preferred treatment for cancer, has adverse effects on the individual’s hearing sensitivity (de Andrade, Khoza-Shangase & Hajat, 2009). This knowledge is particularly important in the management of the paediatric population, as language development, academic progression and social interaction and integration rely heavily on hearing ability (Knight, Kraemer & Neuwelt, 2005). Therefore, healthcare workers, especially in the paediatric oncology team, have a responsibility to inform parents of children undergoing cancer treatment about the ototoxic effects of chemotherapy in this population.

Cancer is defined as the uncontrolled division of abnormal cells which results in tumours (Cooper, 1992). In most cases, these tumours are often not immediately identified; they usually form gradually resulting in an increasing mass of cancerous cells in the body (Cooper, 1992). It should be noted that not only is there diversity in the type of cancers that may be encountered, but there is also diversity in the manifestation of this disease as individual cancers of the same type may present differently (Cooper, 1992). Because of late diagnosis and management, the abnormal cancer cell may enter the system of normally functioning organs and tissues, thus leading to the abnormal functioning of the normally functioning cells (Cooper, 1992).

‘Cancer is a major public health problem in the USA and many other parts of the world. One in four deaths in America is due to cancer’ (Jemal et al., 2008, p. 71). Locally, in South Africa, the burden of disease with regard to cancer appears to be rapidly rising. Adewole et al. (2013) estimated that the burden of cancer is expected to increase to greater than 85% by year 2030. The incidence of cancer in Southern Africa, in particular, appears significantly higher compared with the rest of the continent (Mqoqi et al., 2004). However, the exact statistic regarding paediatric cancer is not readily available, especially in South African public healthcare sectors (Childhood Cancer Foundation of South Africa, 2014), while in the United States, after accidents, cancer is the leading cause of death in paediatrics aged between 1 and 14 (Jemal et al., 2008).

Historically, surgery and radiotherapy were the primary treatment options for cancer until the 1960s when chemist Paul Ehrlich developed and introduced drugs, which he termed chemotherapy, to manage infectious diseases (DeVita & Chu, 2008). In recent times, however, various alternative options are available for the treatment of cancer, inclusive of surgery, radiation therapy and chemotherapy, or, depending on the individual case, a combination of these may be used (Sikara, 2008). According to the Childhood Cancer Foundation of South Africa (2014), cancer within the paediatric population is complex and often occurs in the developing cells within the child’s body. Treatment for these cancers is, therefore, equally complex and thus requires specialised care by paediatric oncologists.

Chemotherapy, as explained by Nygren (2001), is the intravenous or oral administration of a combination of chemicals which kill cells to eliminate or reduce the encountered malignancy, based on the patient’s histology. Chemotherapy involves the administration of cytotoxic drugs, in an attempt to kill off all cells of a cancerous nature (Nygren, 2001). With the use of chemotherapy, there has been an increase in paediatric cancer survival rates currently estimated at 75% – 80% (Rheingans, 2007). Since the primary goal of treatment is to cure cancer and its symptoms, considerations regarding the toxic nature of chemotherapy or the quality of life in paediatrics living with cancer are usually secondary (Wolfe et al., 2000).

Many adverse effects, which occur directly as a result of chemotherapy, have been well-documented in the literature. A large variety of chemotherapeutic agents, such as platinum compounds including cisplatin, carboplatin, nitrogen mustard and bleomycin, are common components in chemotherapeutic regimes and are also strongly associated with hearing loss (de Andrade et al., 2009). Cisplatin, in particular, has been identified as a commonly used, robust chemotherapeutic agent, which often aids in the success of treatment, despite its highly ototoxic nature (de Andrade et al., 2009). According to Knight et al. (2005), cisplatin and carboplatin were absolutely vital in the treatment of a broad spectrum of cancers occurring during childhood. These agents, however, have demonstrated significant ototoxic effects, with literature indicating a particularly high susceptibility to ototoxic hearing loss, as a direct result of exposure to these agents (de Andrade et al., 2009; Gurney et al., 2007), especially in the paediatric population.

Schlauch and Nelson (2009) reported concern that ototoxic drugs cause reductions in the hearing sensitivity of high-frequency sounds. This occurs as a direct result of the damaging effects of the medication on the outer hair cells of the cochlear (Gurney et al., 2007; Schlauch & Nelson, 2009). Although the exact mechanism of the damage is unknown, the end result is generally a bilateral, permanent sensorineural hearing loss, with a 26% – 90% incidence reported in young children, on cumulative doses of cisplatin (Castelon-Martinez et al., 2014). There are sufficient data indicating hearing loss is a long-term side effect resulting from chemotherapy (Gurney et al., 2007; Laverdiere et al., 2005). This sensorineural hearing loss significantly affects language development, verbal abilities and reasoning skills in the paediatric population, regardless of the severity of the hearing loss (Frymark et al., 2010; Gurney et al., 2007). The negative effects of this ototoxic hearing loss on language development in paediatrics need mention as it is usually under reported.

Hearing loss induced by ototoxicity may vary in severity from patient to patient (Frymark et al., 2010) and is unique in the paediatric population, in that it may have grave implications for many other aspects of the child’s life, including educational attainments, speech and language development, and socialisation (Bale, Smith & White, 2005). According to Hoover, Lewis, Moeler, Pitman and Stelmachowitz (2004), specific areas of difficulty as a result of a decline in hearing sensitivity include delays in vocabulary, decreased verbal ability and immature language and reasoning. Results of a study conducted by Bale et al. in 2005 revealed that children with high-frequency hearing losses, which is often characteristic of ototoxicity, present with significant delays in phonological development and, therefore, further delays may be noted in literacy development, as phonological awareness precedes literacy.

It is, therefore, vital that parents of children who undergo chemotherapy are informed about the possible side effects of chemotherapy. Literature states that ‘the principle of informed consent obligates physicians to explain possible side effects when prescribing medications’ (Wells, 2012, p. 1). However, some authors have argued that informing patients about the side effects may cause harm and distress (Benedetti, Lanotte, Lopiano & Colloca, 2007). This distress may manifest though a variety of non-specific symptoms such as psychological distress, poor adherence to treatment, unnecessary consultation and over prescribing medications to treat these affects (Barsky, Saintfort, Rogers & Borus, 2002). However, disclosure or non-disclosure of possible side effects may present challenges ethically, as physicians are bound by the principle of non-maleficence (Wells, 2012). Therefore, Wells (2012) recommended engaging a pragmatic approach in minimising the possible side effect which may arise because of disclosure, while maintaining patient autonomy through contextualised informed consent. The contextualised informed consent involves:

taking into account the possible side effects, the person being treated, and the disease involved to tailor the information provided about medication side effects to provide the most transparency with the least potential harm. (p. 5)

For further discussion on the pros and cons of the contextualised informed consent approach, the reader is directed to Wells (2012). For this study, the authors advocate for the use of this approach where the paediatric oncologists, while the goal is to save lives, should also inform the parents about the possible side effects such as hearing loss that may arise as a result of chemotherapy. The paediatric oncologists also need to consider that with the use of chemotherapy, there is an increase in paediatric cancer survival rates (Rheingans, 2007). Therefore, they should be transparent about the side effects as they may impact on the quality of life of the paediatric population undergoing cancer treatment.

Therefore, the current pilot study seeks to establish whether paediatric oncologists inform parents about the possible ototoxic effects that the paediatric population undergoing chemotherapy may develop. Furthermore, the study also investigates whether parents of children undergoing chemotherapy are aware of these possible ototoxic effects, which may impact on the family’s quality of life and on the affected child’s language development and scholastic performance.

## Method

### Objectives

The aim of this study was to explore whether paediatric oncologists inform parents about the possible ototoxic side effects of chemotherapy, and to explore whether the parents of children undergoing chemotherapy are aware of ototoxic effects of chemotherapy.

### Study design

A quantitative, non-experimental study was conducted to collect data through questionnaires. Two questionnaires, open- and close-ended questionnaires, were conducted to collect data. The first questionnaire (Appendix 1) was tailored for paediatric oncologists and the second questionnaire (Appendix 2) was for the parents. This study forms part of a larger qualitative study with a focus on the quality of information given to mothers of children who are undergoing chemotherapy. Prior to conducting a qualitative study, a quantitative study (this pilot study) through the use of questionnaires was conducted to ascertain whether mothers are aware of the possible hearing loss associated with chemotherapy. On confirming that mothers are aware, a qualitative study was undertaken.

### Sampling strategy

A convenience sampling strategy was employed to recruit 11 paediatric oncologists and 7 parents from two public hospitals in Gauteng.

### Inclusion criteria

Only paediatric oncologists with a minimum of 12 months experience were included in this study to ensure that recruited paediatric oncologists were familiar with protocols observed in the management of paediatrics undergoing chemotherapy treatment.

Only parents of children who have undergone chemotherapy consisting of either cisplatin or carboplatin within the last 2 years were included in the study. The age and the gender of the children were not collected for this study.

### Description of participants

A total of 11 paediatric oncologists were recruited from two public hospitals in Gauteng and 7 parents from the same hospitals were recruited from the inpatient and outpatient paediatric oncology department.

### Data collection tools

**FIGURE 1:**
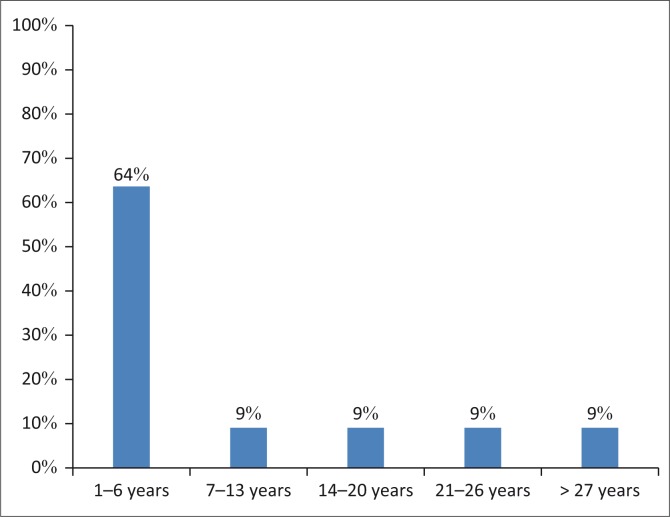
Paediatric oncologists work experience in years (*n* = 11).

Two different questionnaires were administered to the two groups of participants in this study. The questionnaire for the oncologists was a modified version of a questionnaire developed by Khoza-Shangase, Mupawose and Mlangeni (2009). This questionnaire was adapted in order to better fit the context of paediatric oncologists practicing in South Africa. The questionnaire for parents was developed by the researcher after conducting a comprehensive literature review. Both questionnaires were semi-structured and consisted of close- and open-ended questions to enhance the findings of the research and to provide a more comprehensive subject matter (Denscombe, 2010). The questionnaires for the doctors sought to elicit information regarding the years of experience as a paediatric oncologists (Figure 1), their knowledge of the protocols in the management of paediatric cancer patients and the referral rate to audiologist for the management of a possible hearing loss. With regard to the questionnaire designed for parents, the focus was on the length of the period their child has been undergoing chemotherapy treatment, the type of chemotherapy they were receiving, whether they were informed about the effects of chemotherapy on hearing and whether they have noticed any changes on their child’s hearing since undergoing chemotherapy.

### Data collection procedure

Ethical clearance was obtained from the Medical Ethics Board of the University of the Witwatersrand (M150221), as well as from CEOs and head of the departments (HODs; Paediatric Oncology Ward) from the two public hospitals included in the study. Furthermore, an approved copy of the study proposal was forwarded to each hospital. Following approval, questionnaires were distributed to the oncologists and parents for completion via the HODs with the aid of oncology nurses. Each participant was given the information letter, detailing the study as well as consent form. Only participants who signed informed consent forms were included in this study. Participants were aware that they could withdraw from the study at any point without any negative consequences. Personal details of the participants and hospitals were not mentioned in the research report to ensure anonymity. The oncologists were requested to complete the questionnaire regarding their current perceptions concerning ototoxicity protocols in the management of the paediatric population, while the parents were invited to complete a questionnaire exploring length of treatment, hearing status of the child and information received regarding chemotherapy and ototoxicity. Both the administered questionnaires were completed in the absence of the researcher, thereby ensuring confidentiality and anonymity. The completed questionnaires were collected by the researcher in sealed envelopes, from the HODs and nurses.

### Data analysis

Data from the questionnaires were analysed using descriptive statistics to describe specific trends and to summarise measures of some characteristics of the sampled data (Durrheim, 2006). For this study, the responses from both the paediatric oncologists and parents were analysed quantitatively and the results were reported through percentages to indicate the trends and to summarise the findings.

### Validity and reliability

Prior to the commencement of this study, a pilot study was conducted with one paediatric oncologist, who was not included in this study, to assess any potential problems with the data collection tool and to ensure reliability of the study. There were no changes made to the research tool based on the results from the pilot study. A pilot study was not conducted with the parents. Furthermore, in order to achieve a greater reliability, the study was closely observed and executed by both the researcher and the study supervisor who also acted as a peer reviewer throughout the study.

## Results

### Paediatric oncologists

#### Information to parents regarding chemotherapy and ototoxicity

**FIGURE 2:**
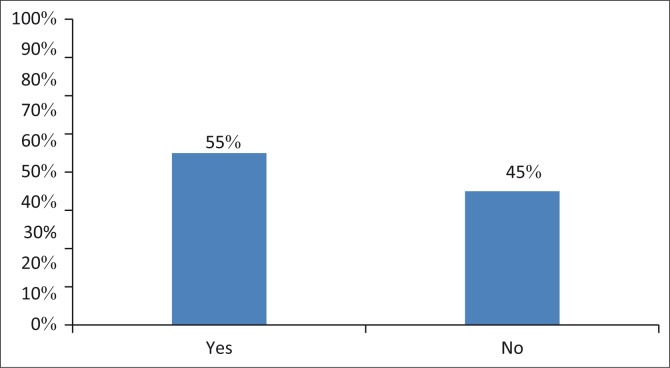
Percentage of paediatric oncologists offering information regarding ototoxicity to parents of children receiving chemotherapy (*n* = 11).

The results of this study indicated that 55% (*n* = 6) of paediatric oncologists offered information regarding the effects of chemotherapy and its impact on hearing, while 45% (5) reported offering no information regarding chemotherapy, despite being aware of its possible negative impact on hearing. A study conducted by McKenna, Collier, and Blake (2009) found that accessibility, support and degree of information offered by healthcare professionals to parents impact on the parent’s confidence when making long-term decisions in the interest of a child undergoing chemotherapy treatment. The finding of this study is some paediatric oncologists provide information to the parents which may guide them when making long-term decisions with regard to the type of treatment for their children. Therefore, the amount and quality of information given to parents regarding ototoxicity and chemotherapy are imperative as this helps guide the treatment process (Figure 2).

Failure to provide parents with information may be counterproductive in empowering and informing parents about early identification and management of any ototoxic hearing loss. Caradini, Cigana, Selistre, Rosito and Brunetto (2007) reported that early detection of potential platinum-based drug–induced ototoxicity may lead to clinical decisions concerning drug dosage, as well as enrolment in early intervention programmes, which may include both the speech therapist and the audiologist. The lack of information rendered to parents by professionals, therefore, undermines the importance of referring patients to audiologists for early intervention programme during or following chemotherapeutic treatment. de Andrade et al. (2009) found that 50% of oncologists in their study presented with a limited awareness regarding the role of the audiologist in patients undergoing cancer treatment. This is concerning as it suggests that paediatric patients are not referred timeously and accordingly for the management of hearing loss during and after chemotherapy treatment.

#### Audiology referrals

**FIGURE 3:**
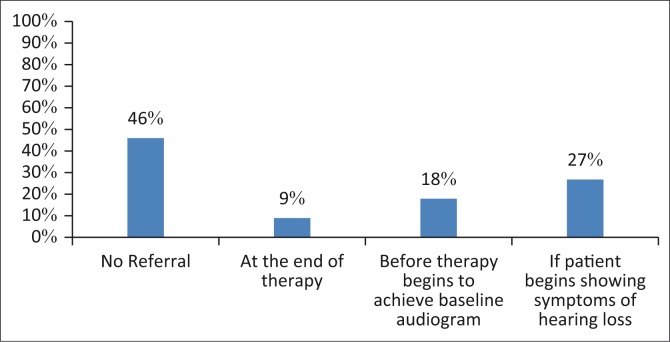
Percentage of paediatric oncologists who refer to audiologists for possible hearing loss management (*n* = 11).

Since 55% (*n* = 6) paediatric oncologists highlighted being aware of the possible chemotherapy effects on hearing, it was crucial to determine whether the paediatric oncologists referred to the audiologists for the assessment of the auditory system before, during and after the administration of chemotherapy on the paediatric population (Figure 3).

The results indicated that 55% (*n* = 6) of participating paediatric oncologists within the study refer to an audiologist during or following the course of treatment, whereas 45% reported never referring to the audiology department (*n* = 5). It is worth noting that these are the same paediatric oncologists who inform parents about the possible effects of chemotherapy on the hearing status. This apparent lack of referrals to audiologists impacts on the early identification and intervention of children who may present with ototoxic hearing loss because of chemotherapy. According to Khoza-Shangase, Barrat and Jonosky (2010), patients generally only notice hearing deficits once a communication difficulty is present, indicating possible ototoxic progressions, as communication difficulties indicate losses in the frequencies responsible for speech. Therefore, these results highlight the need for education on the benefits of early hearing identification and intervention.

Of the six paediatric oncologists, 9% (*n* = 1) indicated referring to the audiologists at the end of therapy, and 18% (*n* = 2) indicated referring for audiological assessment before therapy commences so as to obtain a baseline audiogram. The remaining paediatric oncologists (27%, *n* = 3) reported that they only refer to the audiologist when the patient starts presenting with hearing loss symptoms. These results are concerning as prior to treatment, all patients should be referred to the audiologist to obtain the baseline audiogram and ongoing audiometric evaluations (Vasquez & Mattucci, 2003). These results call for the involvement of audiologists, who are knowledgeable professionals in the prevention, monitoring and management of hearing and hearing-related difficulties because of chemotherapy (Katz, 2002). A study conducted by Bhagat et al. (2010) highlighted the importance of ototoxicity monitoring protocols in patients undergoing chemotherapy treatment in order to screen for any hearing loss symptoms post exposure to known ototoxic agents.

### Parents

#### Type of chemotherapy received by the paediatric patients undergoing chemotherapy

Because of the nature of the study and how data were collected (questionnaires given to the HODs of the Oncology Department, and not directly to the parents), it was not possible to verify the reliability of the responses given by the parents. However, for the qualitative study, this limitation will be addressed as permission to access the patient files has been requested.

**FIGURE 4:**
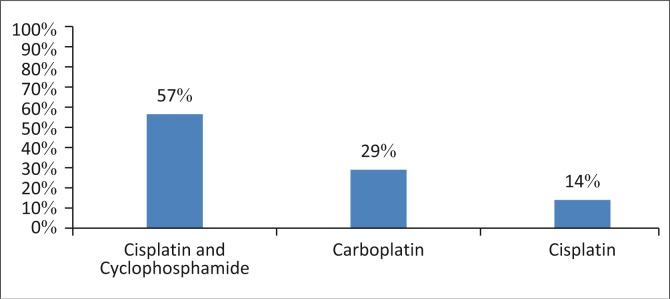
Percentage of children receiving various chemotherapeutic regimens (*n* = 7).

The majority of children (57%, *n* = 4) received a combined dosage of both cisplatin and cyclophosphamide, which are known strong ototoxic agents (Figure 4). Moller, Langguth, DeRidder and Kleinjung (2011) argued that the simultaneous use of multiple ototoxic medications should be avoided whenever clinical circumstances permit, as concomitant use of these may increase a patient’s risk of permanent hearing loss. Also, Caveletti, Fabbrico, Minoia, Frattola and Tredici (1998) stated that alternating chemotherapeutic regimens may be viewed to be advantageous in the treatment of cancer, as these utilise many active agents at one time in the combat of cancerous cells. A small number of children received carboplatin (29%), despite literature noting it to be less ototoxic in nature than other platinum compounds such as cisplatin (Lokich & Anderson, 1998). It must be noted, however, that treatment efficacy profiles between cisplatin and carboplatin differ distinctively based on the type of cancer being treated and, therefore, the type of chemotherapy administered will dependent on the cancer being treated. A small number of participants (14%) were noted to be receiving cisplatin chemotherapy.

#### Information received by parents

**FIGURE 5:**
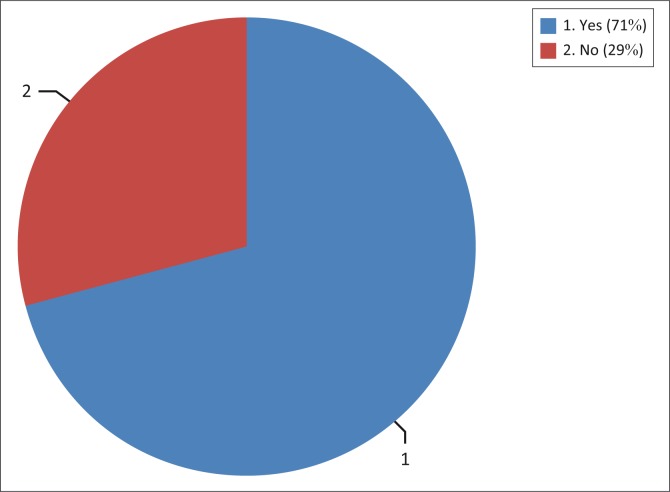
Graph presenting percentage of parents who received information from paediatric oncologists (*n* = 7).

A large majority of parents (71%, *n* = 5) were informed about the possible negative effects of chemotherapy, whereas 29% (*n* = 2) did not receive any information (Figure 5). It is encouraging that the majority of the participants were informed about the possible negative effects of chemotherapy on hearing. McKenna et al. (2009) noted that parents become more involved in decision-making in management of children with cancer when doctors provide adequate information regarding cancer outcomes on paediatrics. This may, therefore, advocate for the role of the audiologist in the public sector with regard to educating people about the possible ototoxic effects of chemotherapy; according to Khoza-Shangase et al. (2010), it is absolutely vital that patients be aware of the effects of treatment on hearing, in order to further pursue management options.

Overall results from the questionnaire administered to the parents appear to indicate that a majority of parents of children receiving platinum-based chemotherapeutic treatment were informed about the possible negative effect of this treatment on hearing before the commencement of therapy.

Furthermore, a majority of parents participating within this study indicated that their primary source of information regarding chemotherapy-related sequel was the medical professionals directly involved with the case. This is a particular point of interest as literature has found that parents tend to rely primarily on the input of their oncologists and medical staff for information regarding treatment, course, side effects and prognosis of the child’s cancer (Massimo, Wiley & Cassari, 2004). This necessitates effective communication of all information between professionals and parents, in order for parents to be properly educated regarding chemotherapy-induced ototoxicity and promote further detection and management where necessary.

## Ethical consideration

Ethical clearance was obtained from the Medical Ethics Board of the University of the Witwatersrand (M150221).

## Discussion

### Limitations of the study

This study comprised only 11 paediatric oncologists and 7 parents of children undergoing chemotherapy treatment. Therefore, the results of this study may not be generalised to a larger population. These results should be seen as preliminary findings of a larger study with a bigger sample size. Furthermore, the percentages used are not of statistical significance as the study comprised a small sample. However, this is a pilot study, hence the small sample size.

Initially, the study sought to engage doctors in the public and private sector. However, during data collection, it was observed that the paediatric oncologists, who participated in this study, although they were recruited from the public sector, are also the same doctors who work in the private sector, thus, lowering the number of sites for data collection.

## Conclusion

The findings of this study are promising as in general it seems that parents are being informed about the ototoxic effects of chemotherapy. Although the number of oncologists who inform parents about possible hearing loss in paediatrics undergoing chemotherapy is still low (only 55%) it is however a step in the right direction. On the contrary, the majority of the parents (71%) indicated being aware of the effects of ototoxic effects, a more encouraging outcome. However, what is concerning about these results is the fact that, as much as parents are aware of the ototoxic effects of chemotherapy, the majority of the children (57%) are receiving a combined dosage of both cisplatin and cyclophosphamide, which are ototoxic in nature (Moller et al. 2011), while a small number of children received carboplatin (29%), despite literature noting it to be less ototoxic in nature than other platinum compounds such as cisplatin (Lokich & Anderson, 1998).

The selection of chemotherapeutic drugs to include in a patient’s regimen is commonly based on the patient’s histology (Nygren, 2001), as each individual’s cancer symptoms may present differently, and therefore necessitate specific chemotherapeutic agents (Cooper, 1992). However, knowing the side effects directly linked to chemotherapy treatment, these results highlight the need for other considerations to be implemented in order to minimise the side effects of chemotherapy in paediatrics who survive cancer.

Some authors have suggested administering low levels of chemotherapeutic agents known to cause hearing loss in children (Knight et al., 2005), the involvement of audiologists in the management of children undergoing chemotherapy (de Andrade et al., 2009; Khoza-Shangase et al. 2010) and educating health professionals involved in the treatment of children diagnosed with cancer (Khoza-Shangase et al., 2010). However, even with these suggestions, the incidence of sensorineural hearing loss has not decreased, especially in developing countries like South Africa (Bale et al., 2005). The results of this study indicated that a small number of paediatric oncologists refer to audiologists for the management of possible high-frequency hearing loss that a child may present with during or post chemotherapy treatment. De Andrade et al. (2009) advocated that the presence of an audiologist within the paediatric oncology team will enhance the management of paediatric patients undergoing chemotherapy, as the audiologist may provide valuable input and expertise in the management of this population.

There is, therefore, a need for conducting further studies, such as the ongoing qualitative study looking at the quality and the mode in which the information is given to the parents to ascertain how parents are informed about the possible side effects of chemotherapy. This mode in which parents are informed about the possible side effects related to chemotherapy is critical, considering that a large number of children are still receiving chemotherapeutic drugs that are directly linked to hearing loss. It is important to establish the quality of information given to the parents to understand if they are made aware of possible options, such as choosing carboplatin, which is less ototoxic, over cisplatin and cyclophosphamide, which are highly ototoxic (de Andrade et al., 2009; Lokich & Anderson, 1998), where possible. This study was concerned with finding out whether parents were informed and aware of the possible hearing loss as a result of their children receiving chemotherapy. Conducting a qualitative study with a focus on the quality and the mode in which the information is given to the parents will be beneficial in informing health professionals about the quality of information given to parents. This will assist in establishing a comprehensive resource for parents of children undergoing chemotherapy.

## Appendix 1

Adapted questionnaire (Khoza-Shangase, Mupawose, & Mlangeni, 2009) addressed to paediatric oncologists.

1. How long have you been practicing in this department at this hospital?
  _______________________________________________________________________________________
  _______________________________________________________________________________________
2. Where have you practiced before this department?
  _______________________________________________________________________________________
  _______________________________________________________________________________________
3. To your knowledge, do chemotherapeutic drugs have any effect on hearing ability?
  **Table:**
  Yes		No		Uncertain	
4. Is there a hearing ototoxicity monitoring programme in place for children receiving chemotherapy?
  **Table:**
  Yes		No	
5. If yes, please provide a brief description?
  _______________________________________________________________________________________
  _______________________________________________________________________________________
  _______________________________________________________________________________________
  _______________________________________________________________________________________
  _______________________________________________________________________________________
6. Is any information offered by you regarding ototoxicity to the parents of the child receiving chemotherapy?
  **Table:**
  Yes		No	
7. If yes, please provide a brief description of this information?
  _______________________________________________________________________________________
  _______________________________________________________________________________________
8. Are you aware of the negative hearing-related sequelae involved, with regard to quality of life issues, which may arise as a result of chemotherapy?
  **Table:**
  Yes		No	
9. If yes, please state whether or not this knowledge generally affects the prescriptive dose of chemotherapy allocated?
  **Table:**
  Yes		No	
10. To your knowledge, what is an audiologist?
  _______________________________________________________________________________________
  _______________________________________________________________________________________
  _______________________________________________________________________________________
  _______________________________________________________________________________________
11. During the course of chemotherapeutic treatment, are paediatric patients ever referred to an audiologist?
  **Table:**
  Yes		No	
12. If yes, please state at what stage is the referral is made?
  _______________________________________________________________________________________
  _______________________________________________________________________________________
  Thank you for your time.

## Appendix 2

Questionnaire addressed to the parents of children who are currently receiving or who have received platinum-based chemotherapy.

1. How long has your child been receiving chemotherapy?
  _______________________________________________________________________________________
2. Prior to the commencement of the treatment, were you provided with any information regarding chemotherapy and hearing? (Please Tick)
  **Table:**
  Yes		No	
3. If yes, please state whether or not you felt this information to be adequate and satisfactory?
  **Table:**
  Yes		No	
4. Please state whether or not you have noticed any changes in your child’s hearing since the beginning of the chemotherapeutic treatment?
  **Table:**
  Yes		No	
  If yes, please explain this?
  _______________________________________________________________________________________
  _______________________________________________________________________________________
  _______________________________________________________________________________________
  _______________________________________________________________________________________
  _______________________________________________________________________________________
5. If any, please list additional sources which you may have consulted for further information regarding chemotherapy-induced sequelae?
  _______________________________________________________________________________________
  _______________________________________________________________________________________
  _______________________________________________________________________________________
  _______________________________________________________________________________________
  _______________________________________________________________________________________
  _______________________________________________________________________________________
  _______________________________________________________________________________________
  _______________________________________________________________________________________
  _______________________________________________________________________________________
6. What are your perceptions of your needs, as a parent, regarding the information given to you by the paediatric oncologist?
  _______________________________________________________________________________________
  _______________________________________________________________________________________
  _______________________________________________________________________________________
  _______________________________________________________________________________________
  Thank you for your time.

## References

[CIT0001] Adewole I., Dangou J., Denny L., Harford J., Bello I. M., Odedina F. et al (2013). Challenges and opportunities in cancer control in Africa: A perspective from the African Organisation for Research and Training in Cancer. *Lancet Oncology*, 14(4), 142–151. https://doi.org/10.1016/S1470-2045(12)70482-5 10.1016/S1470-2045(12)70482-5 23561745

[CIT0002] Bale J., Smith R., & White K (2005). Sensori-neural hearing loss in children. *Lancet*, 365(9462), 879–890. https://doi.org/10.1016/S0140-6736(05)71047-3 15752533 10.1016/S0140-6736(05)71047-3

[CIT0003] Barsky A., Saintfort R., Rogers M., & Borus J (2002). Nonspecific medication side effects and the nocebo phenomenon. *Journal of American Medical Association*, 287(5), 622–627. https://doi.org/10.1001/jama.287.5.622 10.1001/jama.287.5.622 11829702

[CIT0004] Benedetti F., Lanotte M., Lopiano L., & Colloca L (2007). When words are painful: Unraveling the mechanisms of the nocebo effect. *Neuroscience*, 147(2), 260–271. https://doi.org/10.1016/j.neuroscience.2007.02.020 17379417 10.1016/j.neuroscience.2007.02.020

[CIT0005] Bhagat S.P., Bass J.K., White S.T., Qaddoumi I., Wilson M.W., Wu J. et al (2010). Monitoring carboplatin ototoxicity with distortion-product otoacoustic emissions in children with retinoblastoma. *International Journal of Pediatric Otorhinolaryngology*, 74(10), 1156–1163. https://doi.org/10.1016/j.ijporl.2010.07.004 20667604 10.1016/j.ijporl.2010.07.004 PMC4787621

[CIT0006] Caradini P., Cigana L., Selistre S., Rosito L., & Brunetto A (2007). Ototoxicity from cisplatin therapy in childhood cancer. *Journal of Paediatric Oncology*, 29(6), 355–360. https://doi.org/10.1097/MPH.0b013e318059c220 10.1097/MPH.0b013e318059c220 17551394

[CIT0007] Castelon-Martinez O., Jimenez-Mendez R., Rodriquez-Isias F., Fierro-Evans M., Vasques-Gomez B., Medina-Sanson A. et al (2014). Hearing loss in Mexican children treated with cisplatin. *International Journal of Paediatric Otorhinolaryngology*, 78(9), 1456–1460. https://doi.org/10.1016/j.ijporl.2014.06.007 10.1016/j.ijporl.2014.06.007 25037447

[CIT0008] Caveletti G., Fabbrico D., Minoia C., Frattola L., & Tredici G (1998). Carboplatin toxic effect on the peripheral nervous system of the rat. *Annals of Oncology*, 9(4), 443–447. https://doi.org/10.1023/A:1008231925889 9636837 10.1023/a:1008231925889

[CIT0009] Childhood Cancer Foundation of South Africa (2014). Retrieved January 18, 2015, from http://www.choc.org.za/index.html

[CIT0010] Cooper G (1992). *Elements of human cancer*. London: Jones and Bartlett.

[CIT0011] de Andrade V., Khoza-Shangase K., & Hajat F (2009). Perceptions of oncologists at two state hospitals in Gauteng regarding the ototoxic effects of cancer chemotherapy: A pilot study. *African Journal of Pharmacy and Pharmacology*, 3(6), 307–318.

[CIT0012] Denscombe M (2010). *The good research guide: For small-scale social research projects: For small research projects*. London: McGraw Hill Education.

[CIT0013] DeVita V.T., & Chu E (2008). A history of cancer chemotherapy. *Cancer Research*, 68(21), 8643–8653. https://doi.org/10.1158/0008-5472.CAN-07-6611 18974103 10.1158/0008-5472.CAN-07-6611

[CIT0014] Durrheim K (2006). *Basic quantitative analysis*. Cape Town: University of Cape Town press.

[CIT0015] Frymark T., Leech H., Mullen R., Schooling T., Venediktov R., & Wang B (2010). Evidence-Based Systematic Review (EBSR): Drug-induced hearing loss- aminoglycosides In *American Speech Language and Hearing Association* (pp. 1–20). Rockville, MD: National Center for Evidence-Based Practice in Communication Disorders.

[CIT0016] Gurney J.G., Tersak J.M., Ness K.K., Landier W., Matthay K.K., & Schmidt M.L (2007). Hearing loss, quality of life, and academic problems in long-term neuroblastoma survivors: A report from the Children’s Oncology Group. *Pediatrics*, 120(5), e1229–e1236. https://doi.org/10.1542/peds.2007-0178 17974716 10.1542/peds.2007-0178

[CIT0017] Hoover B., Lewis D., Moeler M., Pitman A., & Stelmachowitz P (2004). The importance of high frequency audibility in the speech language development of children with hearing loss. *Otolaryngologyy- Head and Neck Surgery*, 130(5), 556–562. https://doi.org/10.1001/archotol.130.5.556 10.1001/archotol.130.5.556 15148176

[CIT0018] Jemal A., Siegel R., Ward E., Hao Y., Xu J., Murray T. et al (2008). Cancer statistics, 2008. *CA: A Cancer Journal for Clinicians*, 58(2), 71–96. https://doi.org/10.3322/CA.2007.0010 18287387 10.3322/CA.2007.0010

[CIT0019] Katz J (2002). *Handbook of clinical audiology*. Boston, MA: Lippincott Williams and Wilkins.

[CIT0020] Khoza-Shangase K., Barrat J., & Jonosky J (2010). Protocols for early audiology interventions services: Views from early intervention practitioners in a developing country. *South African Journal of Child Health*, 4(4), 100–105.

[CIT0021] Khoza-Shangase K., Mupawose A., & Mlangeni N (2009). Ototoxic effects of tuberculosis treatments: How aware are patients? *African Journal of Pharmacy and Pharmacology*, 3(8), 391–399.

[CIT0022] Knight K., Kraemer D., & Neuwelt E (2005). Ototoxicity in children receiving platinum chemotherapy: Understanding a commonly occurring toxicity that may influence social and academic development. *Journal of Clinical Oncology*, 23(34), 8588–8596. https://doi.org/10.1200/JCO.2004.00.5355 16314621 10.1200/JCO.2004.00.5355

[CIT0023] Laverdiere C., Cheung N.K., Kushner B., Kramer K., Modak S., LaQuaglia M.P. et al (2005). Long-term complications in survivors of advanced stage neuroblastoma. *Pediatrics Blood Cancer*, 45(3), 324–333. https://doi.org/10.1002/pbc.20331 10.1002/pbc.20331 15714447

[CIT0024] Lokich J., & Anderson N (1998). Carboplatin versus cisplatin in solid tumors: An analysis of literature. *Annals of Oncology*, 9(1), 13–21. https://doi.org/10.1023/A:1008215213739 9541678 10.1023/a:1008215213739

[CIT0025] Massimo L., Wiley T., & Cassari E (2004). From informed consent to shared consent: A developing process in paediatric oncology. *The Lancet Oncology*, 5(6), 384–387. https://doi.org/10.1016/S1470-2045(04)01496-2 15172360 10.1016/S1470-2045(04)01496-2

[CIT0026] McKenna K., Collier J., & Blake H (2009). Parental involvement in paediatric cancer treatment decisions. *European Journal of Cancer Care*, 19(5), 621–630. https://doi.org/10.1111/j.1365-2354.2009.01116.x 19807776 10.1111/j.1365-2354.2009.01116.x PMC3178788

[CIT0027] Moller A., Langguth B., DeRidder D., & Kleinjung T (2011). *Textbook of tinnitus*. New York: Springer.

[CIT0028] Mqoqi N., Kellet P., Sitas F., & Jula M (2004). Incidence of histologically diagnosed cancer in South Africa 1998–1999. South Africa: National Cancer Registry

[CIT0029] Nygren P (2001). What is cancer chemotherapy? *Acta Oncologica*, 40(2/3), 166–174. https://doi.org/10.1080/02841860151116204 11441929 10.1080/02841860151116204

[CIT0030] Rheingans I.J (2007). A systematic review of nonpharmacologic adjunctive therapies for symptom management in children with cancer. *Journal of Pediatric Oncology Nursing*, 24(2), 81–94. https://doi.org/10.1177/1043454206298837 17332422 10.1177/1043454206298837

[CIT0031] Schlauch R.S., & Nelson P (2009). *Puretone evaluation*. Baltimore, MD: Lippincott Williams & Wilkins.

[CIT0032] Sikara K (2008). Introduction In Price P., Sikara K., & Lillidge L. (Eds.), *Treatment of cancer* (pp. 3–23). New York: Taylor and Francis Group.

[CIT0033] Vasquez R., & Mattucci K.F (2003). A proposed protocol for monitoring ototoxicity in patients who take cochleo- or vestibulotoxic drugs. *Ear, Nose, and Throat Journal*, 82(3), 181–184. 12696237

[CIT0034] Wells R.E (2012). To tell the truth, the whole truth, may do patients harm: The problem of the nocebo effect for informed consent. *The American Journal of Bioethics*, 12(3), 22–29. https://doi.org/10.1080/15265161.2011.652798 10.1080/15265161.2011.652798 PMC3352765 22416745

[CIT0035] Wolfe J., Grier H.E., Klar N., Levin S.B., Ellenbogen J.M., Salem-Schatz S. et al (2000). Symptoms and suffering at the end of life in children with cancer. *The New England Journal of Medicine*, 342(5), 326–333. https://doi.org/10.1056/NEJM200002033420506 10655532 10.1056/NEJM200002033420506

